# Occupational Health Hazards Among Traffic Police in South Asian Countries: Protocol for a Scoping Review

**DOI:** 10.2196/42239

**Published:** 2023-03-08

**Authors:** Ishrat Jahan, Koustuv Dalal, Md Abdullah Saeed Khan, Archi Mutsuddi, Sabeeha Sultana, Md Utba Rashid, Miah Md Akiful Haque, Mohammad Ali Hossain, Mosharop Hossian, Mohammad Hayatun Nabi, Mohammad Delwer Hossain Hawlader

**Affiliations:** 1 Department of Public Health North South University Dhaka Bangladesh; 2 Public Health Professional Development Society Dhaka Bangladesh; 3 Mid Sweden University Sundsvall Sweden; 4 National Institute of Preventive and Social Medicine Dhaka Bangladesh; 5 International Centre for Diarrhoeal Disease Research, Bangladesh Dhaka Bangladesh; 6 Ibn Sina Medical College Hospital Dhaka Bangladesh

**Keywords:** traffic police, occupational health hazards, occupational injuries, occupational exposures, South Asia, occupational, injury, psychosocial, well-being, prevention, database, policy

## Abstract

**Background:**

Occupational health hazards and injuries are an alarming concern among traffic police. Occupational injuries affect the physical, social, and mental well-being of police personnel, which has various public health implications. The evaluation of occupational health and safety policies and regulations for the traffic police relies on their occupational exposure and health hazard statistics and assessments.

**Objective:**

The purpose of this scoping review is to systematically explore, analyze, and describe relevant findings from all studies conducted on occupational exposure and associated health hazards among traffic police in South Asia.

**Methods:**

The scoping review will include studies that assessed occupational exposure prevalence, types, knowledge, predisposing factors, and prevention strategies. Databases like PubMed, Springer Link, EBSCOhost, the Cochrane library, and Google Scholar will be used to obtain both published and unpublished works in the English language. Relevant gray literature, including governmental and international organization reports, will be examined. After removing duplicates and screening titles and abstracts, the full-text analysis will begin. Arksey and O'Malley's methodology framework for scoping reviews will be followed. According to Preferred Reporting Items for Systematic Reviews and Meta-Analyses Extension for Scoping Reviews, the scoping review will be reported. Two qualified reviewers will independently conduct article screening and data extraction. The extracted data will then be tabulated and accompanied by an explanation to facilitate comprehension. We will extract relevant article results using NVivo (version 10; QSR International) and thematic content analysis. The included articles will be evaluated using the mixed methods appraisal tool (version 2018).

**Results:**

The scoping review will provide insight into how occupational health hazards affect traffic police physically and psychologically in South Asia. The theoretical conceptualization of different aspects of the occupational health of traffic police will emphasize future studies in this region, which will inform policy makers to revise their occupational health and safety policies and principles. It will have implications for taking necessary preventive measures in the future to reduce occupational injuries and fatalities resulting from different types of occupational hazards.

**Conclusions:**

This scoping review will describe the overview of occupational hazards among South Asian traffic police and will provide insights for policy makers to implement changes and to adapt new strategies.

**International Registered Report Identifier (IRRID):**

PRR1-10.2196/42239

## Introduction

Where there is unplanned urbanization, occupational- or work-related health risks related to environmental pollution have become a serious public health problem [1]. Workplace health risks include injuries, illnesses, and diseases that originate from a particular occupation and chronic exposure to certain chemicals or progressive physical actions. According to the International Labour Organization, 2.3 million individuals are injured at work every year. Moreover, each day, more than 6000 individuals die as a consequence of workplace accidents [2]. Occupational risks can cause fatalities and prolonged absences from work. Occupational hazards are estimated to cost the global economy 4% of the gross domestic product each year [3].

Traffic police perform crucial actions in maintaining the country's law, regulations, order, and safety-security. They are actively involved in managing the city's commutation system. They ensure that automobiles may travel freely on the road. Their responsibilities include controlling traffic on cross streets, settling roadside traffic disputes, and collecting penalties for breaking traffic regulations. Traffic police officers are continuously compromising their health for the general public and are more exposed to occupational hazards. Since occupation is a major determinant of health, traffic police personnel face multiple occupational hazards, and it has become a serious public health concern. Thus, duty-bound personnel like traffic police are continuously exposed to occupational health risks and hazards and experience severe related health problems [4]. A Canadian study reported that 42% (n=1500) of working Canadians had hazardous occupational noise exposure and 80% (n=373) of them used to wear hearing protection [5]. They are constantly and concurrently exposed to vehicular pollution, noise from vehicle honking, harsh weather conditions, and interpersonal tension between drivers and pedestrians. Traffic police can also get injured or killed due to car accidents and assaults. These can happen while driving, patrolling, or when police are dealing with violence, particularly in the context of South Asia. The possible occupational hazards for traffic police include respiratory illness, noise-induced hearing loss or impairment, skin diseases brought on by UV radiation, musculoskeletal disorders or backaches, psychological issues, occupational cancer, and other diseases [6].

Traffic police encounter a variety of occupational dangers, as one’s occupation is a major driver of one’s health [7]. A systematic review of 36 global studies among police officers concluded that traffic police officers were exposed to noise-induced hearing loss, dermatitis, cancer, posttraumatic stress disorder, and musculoskeletal disorders [8]. Another systematic review revealed that traffic police officers are one among those who are at risk of developing lung cancer due to environmental exposure to polycyclic aromatic hydrocarbons, benzene, and so forth [3]. According to a European study, air pollution is responsible for 6% of all deaths, whereas vehicular pollution is responsible for 50% of all deaths caused by air pollution [7]. Similarly, noise is one of the world's most common occupational health hazards, and the connection between excessive noise levels and noise-induced hearing loss (NIHL) is widely established. As traffic police officers spend most of their day exposed to noise and air pollution and remain in close proximity to the smoke and fumes emitted from vehicles, they experience air- and noise-induced illness. A cross-sectional study was conducted on the respiratory health status of traffic police in Dhaka, Bangladesh, and reported 84.4% of the respondents had different types of respiratory problems, such as cough, whistling problems, and breathlessness. About 3.1% of the traffic police had been experiencing asthma, and 9.1% of respondents had a history of parental lung disease [9]. A study in Mangaluru city, India, found that the prevalence of respiratory morbidity, eye symptoms, and ear problems were 51.2%, 61.6%, and 47.5%, respectively [4]. Research conducted in Patiala, India, showed that out of 100 traffic policemen, 68% had a frequent cough, 22% had shortness of breath, and 36% had irritation in the respiratory tract [10]. Another study conducted in Gujarat, India, studied the different disease prevalence in traffic police personnel and revealed that about 7% of traffic police personnel experience eye problems, 35.5% from blocked ear sensations, and 51.50 % from mild hearing loss, 13.6% from moderate and 0.90% from severe hearing loss. The most common physical problems include joint problems (62.65%), burning soles (42.37%), back pain (20.3%), and disturbed sleep patterns (16.3%) [11]. A study conducted among traffic police in Kathmandu found that 72.3% of the respondents had a complaint of burning eyes or tearful eyes. Ear ringing was reported by 37.8% of the study subjects [12]. Therefore, skin, respiratory, and eye problems were the most common physical problems seen among traffic police reported in another study in Kathmandu [13]. A Malaysian study reported that traffic police experience hypo- or hyperthermia, skin cancer, depression, lower back pain, asthma, NIHL, and so forth due to occupational exposure to major environmental hazards [14]. A study conducted in Hong Kong has assessed that 80% of traffic constables have reported exposure to environmental tobacco smoke, and the odds ratio of having respiratory systems is 20.4 times more in men as compared to women [15].

While dealing with heavy traffic congestion, traffic police become mentally and physically exhausted, making them susceptible to physical and mental stress, increasing with the duration of employment [6]. A study conducted in Kathmandu, Nepal, revealed a significant prevalence of mental health problems, such as depression (41.3%), anxiety (47%), and stress symptoms (44%), among traffic police officials. This study also reported that smoking and longer working hours were significantly associated with depression, anxiety, and stress [16]. About 73% of traffic police were reported with anxiety, while stress-related problems were identified among 40.6% in another study conducted in Kathmandu [13]. Poor pay, workload, and irregular working hours were stress factors causing psychological hazards among Malaysian traffic police [14].

Cardiovascular disease and cancer risk are also linked to traffic pollution [17]. The leukocyte telomere length might reduce in persons exposed to traffic pollution, resulting in early biological aging and an increased risk of developing coronary heart disease, heart failure, diabetes, cancer, and osteoporosis [18]. The time-weighted average exposure to benzene, a carcinogen, was generally higher for traffic police than indoor workers [19].

Many traffic police officers experience work-related musculoskeletal diseases, particularly in the lower back, neck, and shoulders [20]. Musculoskeletal issues related to work are the leading cause of absenteeism and decreased productivity [21]. Standing in a static posture for lengthy periods exposes them to ergonomic issues [22]. Overall, 12% (n=24) of the traffic police in Nepal were found experiencing varicose veins due to long-standing working hours [23]. Another study in Bengaluru, India, reported 60% of study participants had varicose veins associated with long standing hours [24]. An Indian study on traffic police officers reported disorientation in working schedule, fear of being vulnerable to disease, and pressure of maintaining law and order during the COVID-19 lockdown increased their stress level [25]. Job conflict, lack of autonomy, and organizational policies were found to significantly affect burnout among Islamabad traffic police officials [26].

This implies that traffic enforcers' occupational health and safety (OHS) is a critical public health issue, especially in South Asian countries.

Occupational health hazards may be preventable, and the majority of people are ignorant of them. As a consequence, a safety culture must be developed in the workplace. Occupational infection prevention and control training programs are developed at a local level by hospitals or at a national level by Ministry of Health and Professional Organizations in the United States and Australia [27]. New Delhi traffic police have various preventive measures and safety recommendations. They have extensive safety and skill training, they exercise often to stay fit and limit the risk of injury, they use personal protective equipment or other barriers for the duty, and they learn safe lifting techniques. They follow a recommended shift work pattern to protect themselves from the consequences of continuous long shifting hours. They receive first aid training to combat any emergency situation. Blood pressure checkups, cholesterol tests, and psychiatric screenings are provided as part of the Health Surveillance program to ensure general well-being of traffic police officers in New Delhi. Baseline health assessments are carried out before starting training or deployment to a specialty police duty [28]. According to another study conducted in Ahmedabad, traffic officers standing for a long period of time in hot and humid weather and their traffic intersections are not equipped with shade and existing uniform material for traffic police diminish the effectiveness of sweating. In order to deal with the heat, traffic officers drink lemon water, sugarcane juice, and buttermilk. They cover their faces with a handkerchief, which they also use to protect themselves from smoke from car exhaust [29].

In India, regarding OHS policies, key activities include reviews of the labor administration system at the national and state levels, and conduction of workshops to promote ratification of the International Labour Organization conventions on OHS [30]. Control measures being implied among traffic police through the OHS policy of Malaysia were job rotation, using earplugs if necessary to prevent NIHL, counseling by Religious and Counseling Division of the Royal Malaysian Police to prevent psychological hazards, following self-hygiene practices such as handwashing, using face masks, and so on [14]. In Bhutan, though the OHS laws are in place, the Department of Labour is still incompetent to implement the legislation fully. The situation is impaired resistance from employers and employees to comply with the OHS legislation and to adopt a safety culture [31]. The enforcement of OHS laws is being carried out through inspections, emphasizing on the prevention, protection, and promotion. Although the National OHS policy of Bangladesh has gaps, it includes multiple clauses based on international labor policy such as workplace safety, accident prevention, workplace environment and prevention of hazards, disease prevention, and safeguards. The policy stipulates the need to ensure workplace safety and health protection in the light of international conventions, to develop a strategy and action plan to ensure proper implementation of national laws, and to include OHS issues in the policies and programs of all related ministries and agencies [32].

Therefore, directly or indirectly, traffic police face a wide variety of health hazards at their working environment, namely, physical, biological, psychosocial, chemical, and ergonomic hazards. Thus, duty-bound personnel like traffic police get exposed to various occupational hazards with different work-related injuries or diseases that have profound public health implications. These identified impacts pose severe health and safety problems and require inculcating a safety culture in the workplace. After a careful search, limited studies were found focusing on occupational health hazards among traffic police officers in South Asian countries. However, a few relevant studies regarding OHS practices, and guidelines for traffic police officers were conducted in this region. Hence, the conduct of this scoping review is imperative to explore the knowledge gaps, prevalence, possible risk factors, and control or preventive measures of occupational health hazards among traffic police in South Asia.

The suggested scoping review will include studies conducted on the occupational health of traffic police. It will cover all aspects of health, including the physical, psychological, social, and environmental components in this vulnerable group related to their occupational exposure. Furthermore, risk factors causing these exposures, such as insufficient knowledge and preventive measure, will be explored. This study is directed to come up with a complete evaluation of the evidence on occupational hazards among South Asian traffic police.

We will follow the Preferred Reporting Items for Systematic Reviews and Meta-Analyses Extension for Scoping Reviews (PRISMA-ScR) guidelines, use reliable tools to assess study quality [33,34], and propose a method to query the literature about the occupational exposures and health hazards among South Asian traffic police.

## Methods

### Protocol Design

This scoping review will synthesize all available information by systematic research, selection, and amalgam of current knowledge. The conceptual framework developed by Arksey and O'Malley [35] will be followed to develop this protocol. The development of a scoping review will involve six steps: (1) identifying research question; (2) identifying suitable publication or studies; (3) selection of studies; (4) data charting; (5) collating, summarizing, and reporting results; and (6) quality appraisal.

**Figure 1:**
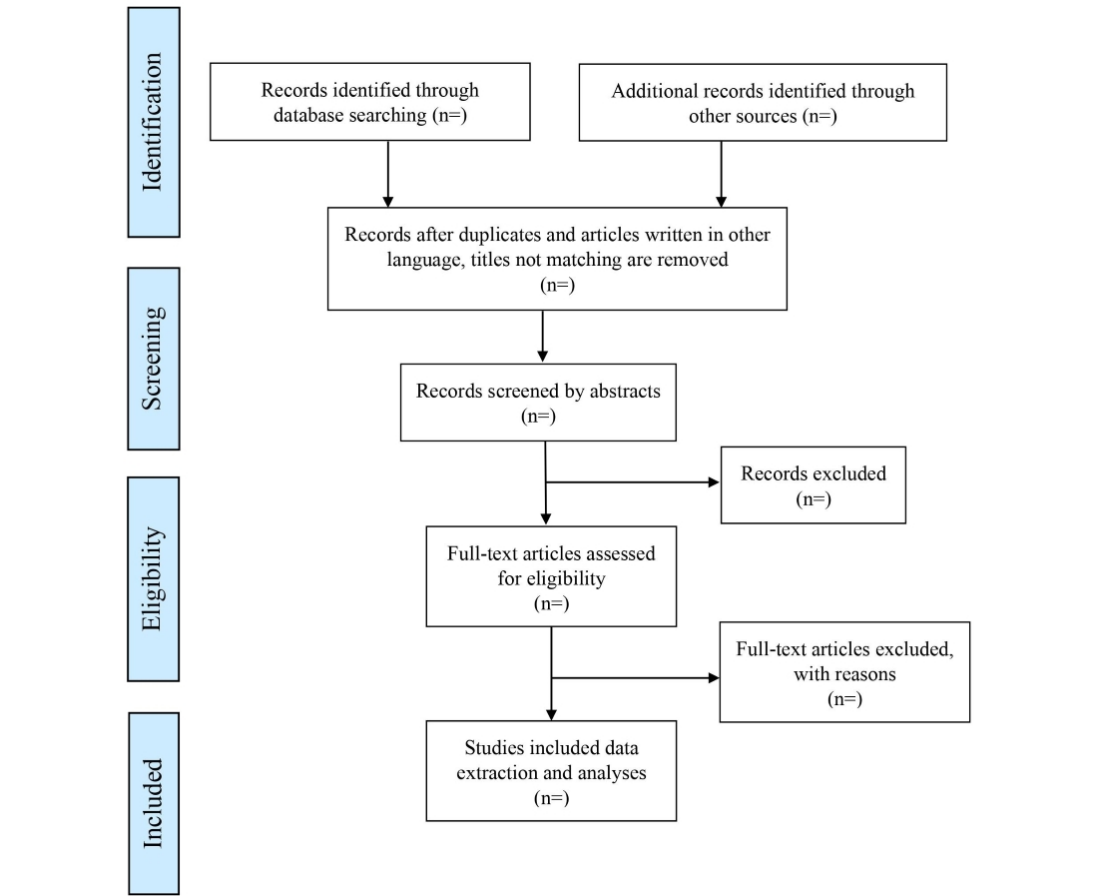
Study selection process.

To assess the quality of the articles incorporated in the review, the mixed methods assessment tool, 2018 will be used [34]. NVivo (version 10) [36] will be used to obtain meaningful outcomes for thematic content analysis. After the observations are input into NVivo, information about occupational health risks among traffic police will be coded. The findings on the number of studies retrieved from the search and the total number of studies excluded at each screening stage guided by the Preferred Items for Systematic Reviews and Meta-Analyses extension for scoping reviews will be reported and is outlined in Figure 1 [33]. Therefore, scoping review will give an outline relevant to our interest, allowing for the clarification of key concepts and the identification of gaps. This scoping review will be used to help us choose an appropriate arena for a future systematic review.

### Eligibility of Research

Studies will be selected according to the population, exposure, and outcomes framework outlined below (Textbox 1).

**Structure to determine eligibility for the research question.**

**Population**

- Traffic police in South Asia

**Exposure**

- Occupational exposure (air pollution, noise pollution, exposure to chemical substances, and negative workplace exposure)

**Outcomes**

- Types
  Physical hazards: noise-induced hearing loss
  Chemical hazards: cancer
  Biological hazards: blood-borne diseases
  Ergonomic hazards: musculoskeletal disorders
  Organizational hazards: burnout
  Psychological hazards: anxiety, depression, insomnia, and posttraumatic stress disorder
- Prevalence
- Level of knowledge
- Predisposing factors or risk factors
- Preventive measures
- Usage of preventive measures

### Identifying Research Questions

Overall, the research questions are as follows: what is the current status of occupational health hazards among South Asian traffic police? Based on this research question, the following questions are specified: (1) what kind of occupational health hazards do traffic police face, and how common are they? (2) What are the traffic police’s understanding or knowledge of the occupational hazards related to their jobs? (3) What are the potential risk factors that put traffic police at risk for health hazards related to their occupation? (4) What are the preventive strategies accessible for use by traffic police to avoid occupational health outcomes? (5) What preventive steps the traffic police might use to avoid workplace health risks or hazards? (6) Is there awareness of various protocols for occupation-related injuries or chronic disease management?

### Identifying Suitable Studies

The scoping review also aims to compare patterns, prevalence, and characteristics of occupational hazards of South Asian countries (India, Pakistan, Bhutan, Bangladesh, Nepal, and Sri Lanka) in which there will be a search for common patterns in regard to occupational health hazards as well as comparison of patterns, characteristics, and prevalence of occupational health hazards by country. We assume that there could be a common culture, common characteristics of the occupational health hazards among the traffic police, and even there could be a similar pattern of environmental exposures as these countries belong to the same subcontinent. On the other hand, there could be variance in prevalence, national policies, and preventive measures in different countries of South Asia. Moreover, most of the studies were conducted on various types of occupational health hazards among traffic police in different countries of South Asia. There are very few studies, which are focused on occupational health hazards of traffic police, thus generalizing findings for South Asia. Therefore, there will be an inclusion of all types of studies conducted on occupational health hazards of traffic police in countries as well as a whole of South Asia to identify the root causes behind prevalent occupational health hazards and also to have a clear picture on the emergence of tailoring any program for traffic police to prevent occupational health hazards.

Another goal of this review is to discover a wide range of literature including published studies, policy reports, newspapers, gray literature, and so on. The following electronic databases will be searched for keywords: PubMed, Springer Link, as well as EBSCO host, the Cochrane library, and Google Scholar (Multimedia Appendix 1). In addition, investigators will also look for appropriate research in gray literature, such as dissertations, as well as reports from governmental and international organizations such as World Health Organization and abstracts from conferences as there are very limited published studies focused on this topic in South Asia available in web-based sources. Finally, investigators will examine the reference lists of the incorporated research for related articles. During database searches and article retrieval, assistance from North-South University library services will be sought. The following are the keywords that will be used to find these databases: traffic police, occupational health hazards, prevalence, types, level of knowledge, predisposing factors or risk factors, preventive measures, and level of use of preventative measures (Multimedia Appendix 1). While searching, Boolean terms (AND, OR) will be used to separate relevant keywords. Additionally, MeSH terms will also help to specify the search. As each database has a distinct search method, the result of database searches will be transferred to the Mendeley library for additional screening of abstracts and complete articles. Every search will have its own specific documentation, which will include all details of the literature such as the search terms, the date of the search, the search engine used, the total number of articles found, and so on Research teams, important stakeholders, and knowledge consumers working in this field, such as health care professionals, university professors, will be polled to choose the search terms that will be used by the search strategy. Search terms or strategy will be changed based on the comments of other partners. In order to avert any risk of bias while selecting publications, the final step of the search process will be conducted in complete anonymity.

### Study Selection

The review approach will consist of two screening steps: (1) A title and an abstract and (2) a full-text evaluation.

Two nonpartisan investigators will review the titles and abstracts of manuscripts to determine whether or not they meet the minimal set of inclusion as well as exclusion criteria.

Inclusion criteria are as follows: (1) studies carried on traffic police; (2) studies conducted on job-related exposures to biological and nonbiological hazards, prevalence, different kinds of hazards, risk factors, knowledge levels, and preventative methods; (3) studies carried out in South Asian countries (Bangladesh, Bhutan, Pakistan, India, Nepal, and Sri Lanka); (4) studies that are based on cohorts, case-control studies, cross-sectional studies, randomized controlled trials, and nonrandomized controlled trials; and (6) scholarly work published in English as well as in other languages with an English translation.

Exclusion criteria are as follows: (1) studies that did not focus on traffic police and occupational health hazards, (2) studies published in languages other than English, and (3) qualitative studies.

Before the actual review of article abstracts, the minimal inclusion and exclusion criteria will be evaluated on a sample of abstracts to make sure they are robust enough to include any studies on occupational exposures among traffic police in South Asia. Articles matching the inclusion criteria will be chosen for a full-text review. Based on the sample review, the inclusion and exclusion criteria will be finalized. In the subsequent step of the process, out of 2 investigators, each will conduct their own independent evaluation of the complete texts of the publication using the study criteria. If reviewers disagree with any article, it will be given a second look, and any further disagreements about the article's eligibility will be discussed with a third investigator. In order to provide a comprehensive record of the review procedure, a flowchart known as the PRISMA-ScR [33] will be constructed (Figure 1).

### Data Charting

The relevant data will be drawn out using an extraction form. Specific details such as year of publication with date, study design, population, study aim, study title, study setting, intervention, proportions, result of the study, key findings pertinent to the study questions, and observations will be extracted and will be used to organize the data of all analyzed studies using a codebook. An extraction sample is provided in Textbox 2. Alterations will be made to the data extraction form on a regular basis. The given data extraction instrument will be then adjusted and adapted as needed while processing data from a source of evidence. Two independent reviewers will extract data from the submitted data separately. Extracted data from the reviewers will then be analyzed and compared. To ensure data accuracy and integrity, any inconsistencies will be addressed. In order to assure data validity and coding, all extracted data will be compiled in a single Excel (Microsoft) document.

**Data charting form.**

- Name of author, date of publication
- Study title
- Objective of the study or research question
- Population
  Sample size
  Characteristics of participants
- Geography of the study
- Study design
- Recruitment setting
- Sampling strategy
- Most relevant and significant findings
- Conclusions

### Collating, Summarizing, and Reporting Results

The objective or goal of the scoping review is to outline the existing evidence and give a comprehensive summary of occupational health risks and hazards among traffic police in South Asian countries. It will not only incorporate the prevalence of hazards but also ensure the usage of preventive strategies. It will also serve as a guide to identify the risk factors. According to Arksey and O’Malley methodology [35], the investigators will use thematic content analysis to extract data linked to the research objectives and code them using NVivo software (version 10) [36]. The full-text papers will subsequently be analyzed for documented evidence on occupational health concerns among traffic police. Through coding, all data on occupational health hazards among traffic police will be collected from the included publications. Lastly, an overall interpretation of how the various concepts relating to the research topic and thematic areas relate to one another will be undertaken.

### Quality Appraisal

For the quality evaluation, evidence from various research will be evaluated. This will be performed to confirm that the research strategy is suitable for the study's objectives and to limit the likelihood of bias. The 2018 version of the mixed methods appraisal tool (MMAT supported by the Canadian Institutes of Health Research) will be applied to evaluate the standard of the study's chosen articles [36]. Using this instrument, researchers will be able to evaluate the objective, methodology, research design, recruitment of study participants, data collection, data analysis technique, description of study outcomes, and authors' debates and conclusions. It will also help to evaluate the quality of primary research. Due to the necessity of making judgments, this technique requires the participation of 2 independent reviewers. Reviewers will adhere to the scoring manual included in the MMAT 2018 version. The overall percentage score for quality will be calculated for each study evaluation, scores below 50% will be regarded as poor and scores between 51% and 75% will be considered average. A score between 76% and 100% will be considered as of good quality.

### Ethical Considerations

This scoping review requires no ethical approval as it does not involve human subjects; yet, it might have some limitations. Limited studies are conducted regarding this topic in this region. Again, this review will only consider published and gray literature written in English.

## Results

A preliminary search of the primary databases has been conducted. Electronic database searches will be completed by October 2022. Disseminating the findings from this scoping review in a scientific peer-reviewed journal has been envisaged. It is anticipated that the theoretical conceptualization on different aspects of occupational health of traffic police will emphasize future studies in this region, which will inform policy makers to revise their OHS policies and principles. Additionally, it will have implications on taking necessary preventive measures in the future to reduce occupational injuries and fatalities resulting from different types of occupational hazards.

## Discussion

### Principal Findings

The scoping review aims to systematically explore, analyze, and describe relevant findings on occupational exposures and their associated health hazards among traffic police in South Asia. However, a few relevant studies regarding OHS practices, policies, and guidelines for traffic policies were conducted in this region. Hence, this scoping review will explore the knowledge gaps, prevalence, possible risk factors, and control or preventive measures of occupational health hazards among traffic police in South Asia.

The scoping review will outline and perform a detailed discussion on occupational exposure focusing on the physical, psychological, and environmental aspects of health affecting traffic police in the South Asian region. The extracted data will be provided in a tabular format and a descriptive summary will explain how the results relate to the scoping review's goals and objectives. The scoping study is expected to shed light on the information and evidence that are currently available on occupational exposure and health hazards for South Asian traffic police, and the summary will also be evaluated, interpreted, and compared considering the existing literature. The review may have far-reaching implications for future projections of occupational exposures and for revising the current policy if needed. It may also reveal different research areas into multiple aspects of occupational exposure and occupational health hazards among traffic police in South Asia.

The findings of this scoping review will be impactful as it will explore in detail the health hazards associated with this profession, and an evidence-based review of the current status of occupational health safety policy and preventive measures as well as interventions will be explained.

The study will explain the need for occupational safety and health strategy for traffic police with the overall vision of providing a healthy, safe, and productive working environment, which should be implemented by all the relevant agencies such as government, industry, and general public. This study will inform policy as well as management principles and interventions. When a strategy on the OHS of traffic police is well developed and implemented, there will be effectiveness on how the traffic police keep themselves safe from occupational health hazards.

### Strengths and Limitations to the Scoping Review

This scoping review will describe its findings in accordance with the Preferred Items for Systematic Reviews and Meta-Analyses Extension for Scoping Reviews guidelines. Five electronic databases and gray literature, such as unpublished theses and dissertations will be used as a primary source of relevant studies. All articles will be screened by 2 independent researchers using a minimum set of inclusion and exclusion criteria. The study will evaluate the quality of included studies in accordance with an established MMAT. However, there is a possibility to overlook some relevant studies as only English-language publications will be considered and reviewed.

## Appendix

Multimedia Appendix 1 Data search strategy.

## Abbreviations

MMAT: mixed methods appraisal tool
NIHL: noise-induced hearing loss
OHS: occupational health and safety
PRISMA-ScR: Preferred Reporting Items for Systematic Review and Meta-Analysis extension for scoping reviews
