# Supplementary material for: Occupational Health Hazards Among Traffic Police in South Asian Countries: Protocol for a Scoping Review
Source: JMIR Res Protoc. 2023 Mar 8;12:e42239. doi: 10.2196/42239 (PMC10034605; doi:10.2196/42239)
Supplement: Multimedia Appendix 1 [file resprot_v12i1e42239_app1.docx]

**Multimedia Appendix 1. Data search strategy.**

(((((((((((("occupant"[All Fields] OR "occupant s"[All Fields] OR "occupants"[All Fields] OR "occupational"[All Fields] OR "occupations"[MeSH Terms] OR "occupations"[All Fields] OR "occupation"[All Fields] OR ("professional"[All Fields] OR "professional s"[All Fields] OR "professionalism"[MeSH Terms] OR "professionalism"[All Fields] OR "professionality"[All Fields] OR "professionalization"[All Fields] OR "professionalize"[All Fields] OR "professionalized"[All Fields] OR "professionalizing"[All Fields] OR "professionally"[All Fields] OR "professionals"[All Fields])) AND ("hazard"[All Fields] OR "hazard s"[All Fields] OR "hazardous"[All Fields] OR "hazardously"[All Fields] OR "hazardousness"[All Fields] OR "hazards"[All Fields])) OR ("hazard"[All Fields] OR "hazard s"[All Fields] OR "hazardous"[All Fields] OR "hazardously"[All Fields] OR "hazardousness"[All Fields] OR "hazards"[All Fields]) OR ("risk"[MeSH Terms] OR "risk"[All Fields])) AND (("occupational health"[MeSH Terms] OR ("occupational"[All Fields] AND "health"[All Fields]) OR "occupational health"[All Fields]) AND ("hazard"[All Fields] OR "hazard s"[All Fields] OR "hazardous"[All Fields] OR "hazardously"[All Fields] OR "hazardousness"[All Fields] OR "hazards"[All Fields])) AND "traffic police"[Title/Abstract] AND ("epidemiology"[MeSH Subheading] OR "epidemiology"[All Fields] OR "prevalence"[All Fields] OR "prevalence"[MeSH Terms] OR "prevalance"[All Fields] OR "prevalences"[All Fields] OR "prevalence s"[All Fields] OR "prevalent"[All Fields] OR "prevalently"[All Fields] OR "prevalents"[All Fields]) AND "types"[All Fields] AND ("risk factors"[MeSH Terms] OR ("risk"[All Fields] AND "factors"[All Fields]) OR "risk factors"[All Fields])) OR "risk factors"[MeSH Terms] OR "causality"[MeSH Terms] OR ("causality"[MeSH Terms] OR "causality"[All Fields] OR ("predisposing"[All Fields] AND "factors"[All Fields]) OR "predisposing factors"[All Fields])) AND (("level"[All Fields] OR "levels"[All Fields]) AND ("knowledge"[MeSH Terms] OR "knowledge"[All Fields] OR "knowledge s"[All Fields] OR "knowledgeability"[All Fields] OR "knowledgeable"[All Fields] OR "knowledgeably"[All Fields] OR "knowledges"[All Fields]))) OR ("knowledge"[MeSH Terms] OR "knowledge"[All Fields] OR "knowledge s"[All Fields] OR "knowledgeability"[All Fields] OR "knowledgeable"[All Fields] OR "knowledgeably"[All Fields] OR "knowledges"[All Fields])) AND ("prevention and control"[MeSH Subheading] OR ("prevention"[All Fields] AND "control"[All Fields]) OR "prevention and control"[All Fields] OR ("preventive"[All Fields] AND "measures"[All Fields]) OR "preventive measures"[All Fields])) OR (("prevent"[All Fields] OR "preventability"[All Fields] OR "preventable"[All Fields] OR "preventative"[All Fields] OR "preventatively"[All Fields] OR "preventatives"[All Fields] OR "prevented"[All Fields] OR "preventing"[All Fields] OR "prevention and control"[MeSH Subheading] OR ("prevention"[All Fields] AND "control"[All Fields]) OR "prevention and control"[All Fields] OR "prevention"[All Fields] OR "prevention s"[All Fields] OR "preventions"[All Fields] OR "preventive"[All Fields] OR "preventively"[All Fields] OR "preventives"[All Fields] OR "prevents"[All Fields]) AND "weights and measures"[MeSH Terms]) OR ("precautionary"[All Fields] AND ("measurability"[All Fields] OR "measurable"[All Fields] OR "measurably"[All Fields] OR "measure s"[All Fields] OR "measureable"[All Fields] OR "measured"[All Fields] OR "measurement"[All Fields] OR "measurement s"[All Fields] OR "measurements"[All Fields] OR "measurer"[All Fields] OR "measurers"[All Fields] OR "measuring"[All Fields] OR "measurings"[All Fields] OR "measurement"[All Fields] OR "measurements"[All Fields] OR "weights and measures"[MeSH Terms] OR ("weights"[All Fields] AND "measures"[All Fields]) OR "weights and measures"[All Fields] OR "measure"[All Fields] OR "measures"[All Fields]))) AND (("level"[All Fields] OR "levels"[All Fields]) AND ("statistics and numerical data"[MeSH Subheading] OR ("statistics"[All Fields] AND "numerical"[All Fields] AND "data"[All Fields]) OR "statistics and numerical data"[All Fields] OR "utilization"[All Fields] OR "utilisation"[All Fields] OR "utilisations"[All Fields] OR "utilise"[All Fields] OR "utilised"[All Fields] OR "utilises"[All Fields] OR "utilising"[All Fields] OR "utilities"[All Fields] OR "utility"[All Fields] OR "utilizations"[All Fields] OR "utilize"[All Fields] OR "utilized"[All Fields] OR "utilizer"[All Fields] OR "utilizers"[All Fields] OR "utilizes"[All Fields] OR "utilizing"[All Fields]))) OR (("level"[All Fields] OR "levels"[All Fields]) AND ("implementability"[All Fields] OR "implementable"[All Fields] OR "implementation"[All Fields] OR "implementation s"[All Fields] OR "implementational"[All Fields] OR "implementations"[All Fields] OR "implementer"[All Fields] OR "implementers"[All Fields] OR "implemention"[All Fields]))) AND ("Sub"[All Fields] AND ("continent"[All Fields] OR "continent s"[All Fields] OR "continents"[All Fields]))) OR ("south asia"[Journal] OR ("south"[All Fields] AND "asia"[All Fields]) OR "south asia"[All Fields]
